# First principles computation of composition dependent elastic constants of omega in titanium alloys: implications on mechanical behavior

**DOI:** 10.1038/s41598-021-91594-5

**Published:** 2021-06-07

**Authors:** R. Salloom, S. A. Mantri, R. Banerjee, S. G. Srinivasan

**Affiliations:** 1 Department of Materials Science and Engineering, University of North Texas, Denton, TX 76203 USA; 2 Center for Agile and Adaptive Additive Manufacturing, University of North Texas, Denton, TX 76203 USA; 3 Advanced Materials and Manufacturing Processes Institute, UNT, Denton, TX 76203 USA

**Keywords:** Atomistic models, Computational methods, Mechanical properties, Metals and alloys

## Abstract

For decades the poor mechanical properties of Ti alloys were attributed to the intrinsic brittleness of the hexagonal ω-phase that has fewer than 5-independent slip systems. We contradict this conventional wisdom by coupling first-principles and cluster expansion calculations with experiments. We show that the elastic properties of the ω-phase can be systematically varied as a function of its composition to enhance both the ductility and strength of the Ti-alloy. Studies with five prototypical β-stabilizer solutes (Nb, Ta, V, Mo, and W) show that increasing β-stabilizer concentration destabilizes the ω-phase, in agreement with experiments. The Young’s modulus of ω-phase also decreased at larger concentration of β-stabilizers. Within the region of ω-phase stability, addition of Nb, Ta, and V (Group-V elements) decreased Young’s modulus more steeply compared to Mo and W (Group-VI elements) additions. The higher values of Young’s modulus of Ti–W and Ti–Mo binaries is related to the stronger stabilization of ω-phase due to the higher number of valence electrons. Density of states (DOS) calculations also revealed a stronger covalent bonding in the ω-phase compared to a metallic bonding in β-phase, and indicate that alloying is a promising route to enhance the ω-phase’s ductility. Overall, the mechanical properties of ω-phase predicted by our calculations agree well with the available experiments. Importantly, our study reveals that ω precipitates are not intrinsically embrittling and detrimental, and that we can create Ti-alloys with both good ductility and strength by tailoring ω precipitates' composition instead of completely eliminating them.

## Introduction

That the presence of the w-phase within the β (body centered cubic or BCC) matrix causes embrittlement of Ti alloys is considered a *fait accompli*. However, what is not often recognized is that the type and nature of the w precipitates, plays a dominant role in determining the overall deformation behavior of the alloy. Metastable β titanium alloys, quenched from the high temperature single β phase-field, often exhibit *athermal* or *quenched-in w* precipitates that inherit the composition of the parent β matrix^1,2^. These precipitates often co-exist with α, αʹ, and α″ phases. These *athermal-ω* precipitates, within the β matrix, are typically not detrimental to plasticity if their volume fraction is not too high, and results in alloys that are ductile and often exhibit good strain-hardenability. Annealing such a quenched system, however, easily causes a compositional partitioning between the β and ω phases^3^. The resulting precipitates are now called *isothermal ω* and they correlate with a larger yield strength and a severe loss of ductility. The underlying causes of such dramatic variations in properties are not well understood.

The attractiveness of metastable β-Ti alloys is in its versatility and the wide range of mechanical properties that can be adjusted via alloying, and thermo-mechanical treatments^4,5^. In addition to superelastic and shape memory effects, Ti alloys can exhibit high ductility, transformation induced plasticity (TRIP), and twining induced plasticity (TWIP) effects, which can be achieved through the coexistence and reversable transformation between β and α″ phases^6,7^. Also, low elastic modulus values can be obtained in Ti alloys through tuning β-stabilizers concentration to near the lower limit of β-phase stability. Values corresponding to an average number of valence electrons per atom (e/a) of ~ 4.25 creates a Ti alloy with Young’s modulus close to human bone^8,9^. However, Ti alloys can also be brittle, and the embrittlement of Ti alloys has often been attributed to the presence of the metastable hexagonal ω-phase, which offers limited slip systems for deformation^1^.

The ω-phase can precipitate in metastable β-Ti alloys upon water quenching from β-phase region or subsequent low temperature aging treatment. Although the precipitation hardening due to ω-phase can be significant, its utilization to strengthen Ti alloys is restricted due to its detrimental brittleness. Therefore, its presence is avoided in the interest of attaining a good ductility. This has driven systematic studies on the formation, phase morphologies, and mechanical properties of β + ω microstructures in metastable b alloys^3,10,11^. Recent studies on the deformation behavior in Ti alloys^12,13^ have correlated elemental partitioning within β and ω phases to changes in their deformation modes from twinning to slip. A high ductility induced by deformation twinning was observed when compositionally congruent ω-phase precipitates form under quenching. A slip deformation mode was also observed upon aging and elemental partitioning of ω-phase containing Ti alloy, which showed a lower ductility than the quenched alloy. Such studies have also revealed that long-time aging creates high-volume fraction of embrittling ω particles. On the contrary, a short-time, low temperature aging produced an ω particle containing ductile Ti–12 wt% Mo (~ 6.5 at.% Mo) alloy, which exhibited a significant increase in strength without significant ductility reduction^14^. Note that this aging treatment had only a minor effect on ω particles volume fraction. This observation was attributed to the chemical partitioning of the solutes between β and ω phases during aging, and was experimentally observed in Ti–V alloy^15^ and later in Ti–12 wt% Mo by Lai et. Al^16^. This urges the need to study the effect of chemical composition on the intrinsic mechanical properties of the ω phase.

While the effect of many alloying elements on β-phase stability and elastic properties are well documented^7,8,17–20^, there is little knowledge on how such β-stabilizers’ influence the elastic properties of the ω-phase^4,21–23^. This experimental limitation is related to the difficulty in preparing ω-phase single crystals. Tane et al.^21^ measured the elastic constants of an aggregate of ω-phase crystals prepared by high pressure torsion process and found that the Young’s modulus and shear moduli of ω-phase is higher than those of β and α phases. However, no such studies have explored the effect of β-stabilizers on the phase stability and elastic constants of ω-phase due to intractable experimental challenges. This is because only two-phase β + ω microstructure forms in Ti-alloys at ambient pressure conditions. Furthermore, the ω precipitates are typically less than tens of nanometers in size and embedded within the β matrix. Lastly, single phase ω is stable at high pressures, which makes experimental measurement of its elastic constants challenging. Therefore, to address our problem, we computed elastic constants of ω as a function of the composition of β stabilizing elements using accurate DFT computations. Our coupling of DFT and experiments provided a powerful approach to answer the long-standing question on ductility of the ω-phase.

The rapidly emerging field of Integrated Computational Materials Engineering (ICME) promises to revolutionize the design new engineering materials. It links different length scales, or knowledge nodes to design a microstructure that meets specific applications requirements. Building effective quantitative models through this approach requires extensive knowledge about different materials properties and thermodynamics. It is well known that there is a direct correlation between elastic constants and strength of any metallic phase^8,24^. Therefore, Studying the phase stability and calculating the elastic properties of Ti–X (X = W, Mo, V, Ta, and Nb) alloys will provide essential inputs to populate the knowledge base and help build effective quantitative models. This study is our effort at understanding of metastable β-Ti alloys towards developing a better functional and biomedical Ti alloys with low Young’s modulus and high ductility.

In this manuscript, we conclusively show that *ω* precipitates are not typically brittle and correlate the rejection of β-stabilizers from isothermal ω precipitates with the associated substantial change in their elastic constants and increases to their average shear modulus. That, the isothermal ω precipitates, when present in sufficient density, are harder to shear. So, during plastic deformation, the β matrix will be relatively easy to deform while its embedded *isothermal* ω precipitates do not. This will create strain incompatibility at the precipitate/matrix interface, and also increase the yield strength and reduce ductility. This incompatibility in due course will also cause dislocation pileup at precipitate/matrix interface and eventually cause the precipitates to crack followed by macroscopic fracture of the sample. Therefore, the effect of ω composition on shear modulus and the resistance to shearing is important in designing ductile yet strong metastable β-Ti alloys. The present study reveals, for the first time, how the β stabilizer composition of the ω precipitates influence its elastic properties, and thereby their deformability. This provides an atomistic insight into previously reported experiments on the discrepant effect of athermal versus isothermal ω precipitates on the ductility of titanium alloys.

Specifically, the current work investigates the effect of β-stabilizers on the formation and the elastic properties of ω-phase using first principles calculations and experimental work. Different concentrations of β-stabilizers were used to analyze their effect on the β → ω phase transformation. The starting configurational models for the different concentrations were chosen using the cluster expansion method^25^. These same models were also utilized to calculate the elastic properties of the ω phase. The ω-phase stability and Young’s modulus decreased with increasing content of β-stabilizers. This trend was related to the change in the bonding behavior between the Ti atoms and the β-stabilizing atoms. Our results also indicated that the effect of ω-phase on the mechanical properties can be tuned and exploited to develop better Ti alloys and, importantly, concludes that the ω-phase need not always embrittle Ti-alloys as is currently believed. The experimental work investigates the effect of ω-phase solute content on the mechanical properties of Ti-Mo alloy. The microstructure characterization of the solutionized and annealed alloys revealed Mo partitioning toward β matrix, and revealed a decrease in ductility of the alloy at lower Mo contents in ω-phase precipitates.

## Simulation methodology

The Vienna Ab Initio Simulation Package (VASP)^26,27^ was used to perform the density functional theory (DFT) calculations. The Kohn–Sham equations of DFT were solved with projector-augmented-wave (PAW) potentials and wave functions^26^. Electron–electron exchange and correlation effects were described by the generalized gradient approximation (GGA) with the functional form proposed by Perdew–Burke–Ernzerhof (PBE)^27^. Careful convergence calculations yielded a plane-wave kinetic energy cutoff of 500 eV. A convergence accuracy of 10^–5^ eV was used for the self-consistent electronic minimization. Monkhorst–Pack k-point meshes with a spacing of 15 × 15 × 15 Å^−1^ were used in conjunction with Methfessel–Paxton integration scheme using a smearing width of 0.2 eV. Applying these settings give energy accuracy of 1 me V/atom for our calculations.

A crucial prerequisite for first principles calculations is a reliable configurational model where the distribution of solute atoms is near the most stable configuration. To achieve this goal for the binary Ti-X (W, Mo, V, Ta, and Nb) alloys, the Cluster Expansion (CE) method implemented in a UNiversal CLuster Expansion code (UNCLE) was used to systematically search for the optimal random configurations that give thermodynamically stable alloys at T = 0 K^25,28^. The CE calculations were performed for the solid solution β-phase of our binary Ti-X alloys across a wide composition range using a bcc supercell with 16 atomic sites. To establish the cluster interaction parameters, the DFT total energies were calculated for 167, 201, 152, 160, 190 different training-set structures for Ti–W, Ti–Mo, Ti–V, Ti–Ta, and Ti–Nb respectively. Our DFT simulations fully relaxed the atomic positions, supercell volume, and supercell shape to obtain suitable equilibrium configurations.

The minimum energy pathways (MEPs) for β → ω transformation was calculated using the generalized solid state nudged elastic band (SSNEB) as implemented in the VTST package^29,30^. The atomic positions and cell degree of freedom (DOF) were relaxed simultaneously. A linear interpolation of atomic positions and cell vectors was used to guess the initial β → ω transformation path. Five intermediate images were used and the end point β and ω structures were fully relaxed with respect to cell parameters and atomic positions. Also, the forces were optimized below 10^–3^ eV Å^−1^ on each image along the MEPs. Note that a direct full optimization of the volume and internal degree of freedom for the end points structures with low content of β-stabilizer caused a large distortion to the lattice and its symmetry, which created a more stable low temperature phase or other metastable phases. Therefore, the relaxation for structures with low β-stabilizer content was performed over two stages. The first stage minimized the volume and internal degree of freedom with a force threshold of 0.2 eV/Å while the second stage used the same minimization scheme but with a lower force threshold of 0.02 eV/Å.

The six elastic constants of the single crystal hexagonal ω-phase (c_11,_ c_12_, c_13_, c_33_, c_44_, and c_66_) were calculated by applying ± 0.01 strains and measuring the response stress tensors. The polycrystalline elastic moduli were calculated using the Voigt–Reuss–Hill approximation^31^. The isotropic polycrystalline elastic moduli can be taken as an average of the single-crystal elastic constants. The theoretical upper bounds of isotropic polycrystalline bulk modulus *B* and shear modulus *G* are given by Voigt in terms of single crystal elastic constants ${c}_{ij}$ as^32^:

1 $${B}_{V}=\frac{1}{9}[{c}_{11}+{c}_{22}+{c}_{33}+2\left({c}_{12}+{c}_{13}+{c}_{23}\right)]$$

2 $${G}_{V}= \frac{1}{15}[{c}_{11}+{c}_{22}+ {c}_{33}+3\left({c}_{44}+{c}_{55}+{c}_{66}\right)-\left({c}_{12}+{c}_{13}+{c}_{23}\right)]$$

And the lower bounds are given by Reuss as follows^32^:

3 $${B}_{R }=\frac{1}{{s}_{11}+{s}_{22}+{s}_{33}+2({s}_{12}+{s}_{23}+{s}_{13})}$$

4 $${G}_{R }=\frac{15}{{4(s}_{11}+{s}_{22}+{s}_{33}-{s}_{12}-{s}_{23}-{s}_{13})+3({s}_{44}+{s}_{55}+{s}_{66})}$$

Here $${s}_{ij}$$ are the elastic compliance constants, which are the components of the inverse elastic constant matrix. The predicted isotropic polycrystalline bulk modulus and shear modulus can be obtained as *B* = ($${B}_{R }+{B}_{V})/2$$ and *G* = ($${G}_{R}+{G}_{V})/2$$*,* respectively. Young’s modulus and Poisson’s ratio are *Y* = $$9BG/(3B+G)$$ and *υ* = $$(3B/2-G)/(3B+G)$$, respectively^33^.

## Experimental procedure

Ti–19 at.%V and Ti–6.5 at.% Mo alloys were prepared using an electric arc furnace. ~ 50-g ingots were prepared from pure Ti, V, and Mo pellets. The Ti–V ingot was flipped and remelted four times to ensure homogenous melting. The sample was cut to small specimens and β-solutionized at 900 °C for 30 min followed by water quenching. The β-solutionized samples were then annealed at 300 °C for 30 min to precipitate well developed ω-phase particles and stimulate compositional partitioning between β and ω phases. Similarly, the Ti–Mo sample was also β-solutionized at 900 °C for 30 min followed by water quenching. Unlike the Ti–V sample, the samples for Ti-Mo were annealed at 475 °C for 48 h. Transmission electron microscopy (TEM, FEI Tecnai F20-FEG TEM operating at 200 kV) was used for microstructure characterization. Compositional analysis at the atomic scale was conducted using 3D atom probe tomography (APT) in a local electrode atom probe (LEAP) 3000 × HR system from Cameca Inc. APT tips and TEM samples were prepared using Focused Ion Beam (FIB) technique with FEI Nova 200 NanoLab dual beam system. EDM machine was used to cut the tensile test dog bone shaped samples with a gauge length of 3 mm, width of 1 mm and thickness of 0.4–0.6 mm and used for mechanical testing. A uniaxial tensile test was carried out with a strain rate of 10^–3^ s^−1^ at room temperature. A total of 4 samples were tested for each condition and the sample showing the values closest to the average are shown here.

## Results and discussion

### Cluster expansion calculations and configurational models

**Figure 1:**
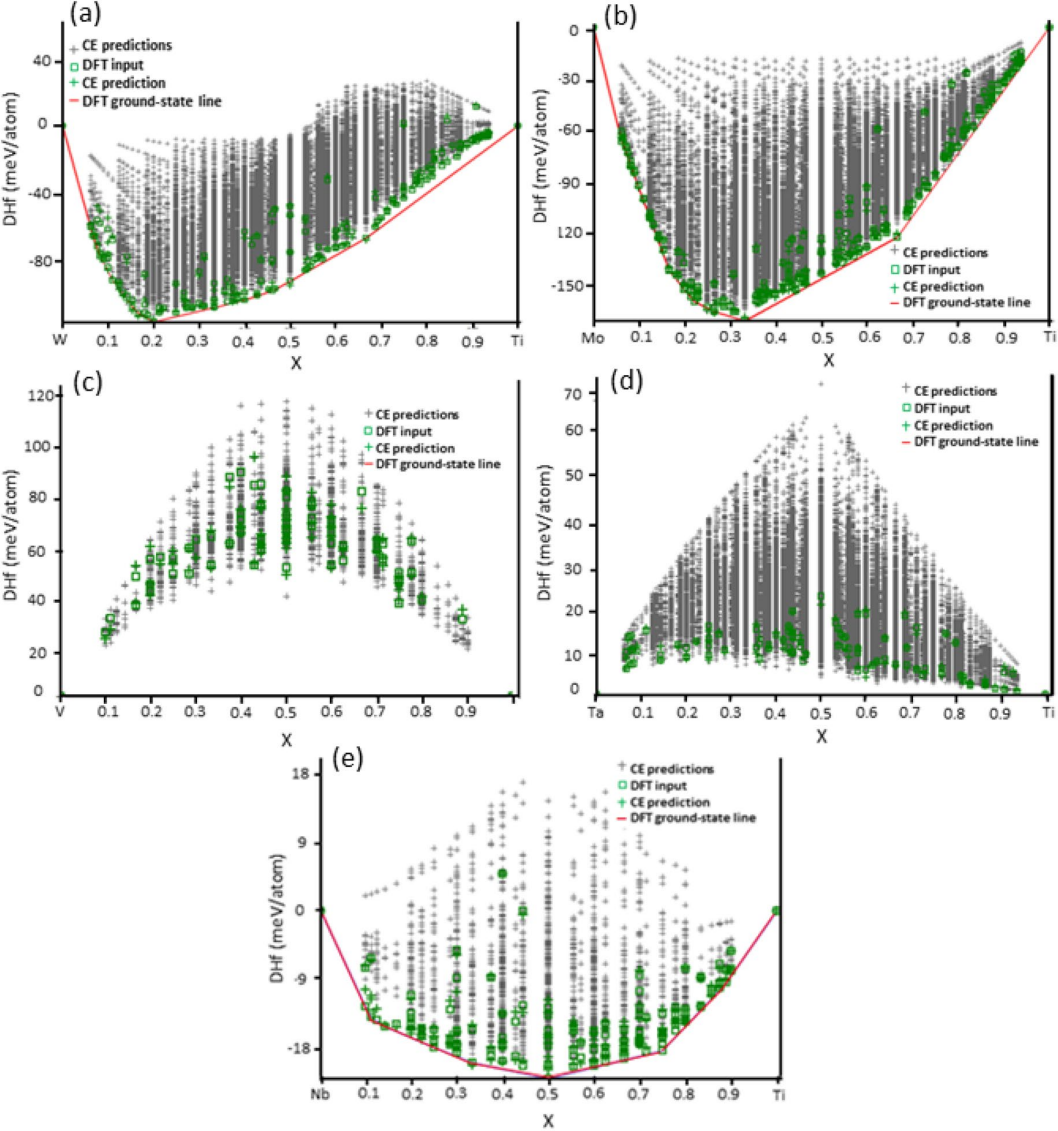
The convex hull plots of binary Ti-X (X = W, Mo, V, Ta, and Nb) β-phase alloys obtained using cluster expansion method. (**a**) Ti–W (**b**) Ti-Mo (**c**) Ti-V (**d**) Ti-Ta and (**e**) Ti-Nb alloys. The CE predictions (grey symbols) represent the structure space within which the ground state structures are identified, while the red CE entries represent the CE training-set structures for which DFT energies are calculated.

The cluster expansion (CE) results are represented by a ground state phase diagram whose X and Y axes respectively represent composition and free energy as shown in Fig. 1 for our prototypical alloys. The ground state search for bcc solid solution structures included all the possible stable configurations within a 16-atom supercell. It can be seen that Ti–V and Ti–Ta binaries exhibit a positive enthalpy of mixing for all the structures in the ground state diagram. This indicates a phase separation tendency within the β-phase of these two binary systems, in agreement with experiments^34^. On the other hand, the ground state diagrams for Ti–W, Ti–Mo, and Ti–Nb show negative formation energies for all convex hull structures, indicating a homogenous mixing of Ti and these elements. However, some previous CE ground state diagrams do not agree with our results for Ti–Nb system. Calculations of Ravi et al.^35^ showed positive formation energy for Ti–Nb. Another study reported that Ti–Nb ground state diagram does not show a separation tendency^36^. This discrepancy may be attributed to using of 6 or fewer atoms per unit cell used by Ravi et al.^35^ for the ground state search, which reduces the number of atomic configurations that can be included within the training-set, hence affecting its output.

Tables 1, 2, 3, 4 and 5 list the stable structures, symmetries, compositions, and schematic representations used for our binary Ti–X (X = W, Mo, V, Ta, and Nb) systems. Here we assume that the ordered structures are representative models for the β-phase. However, we do not imply that Ti forms intermetallic compounds with these β-stabilizing elements. For a specific composition there are many structures lying on the vertical line of the convex hull with small energy difference between them. For example, for Ti–12.5 at.% Mo, the structure closest to the convex hull has Im-3m symmetry as shown in Table 1, and the structure just vertically over it with the same composition has a symmetry of Pm. The energy difference between these two structures is 1 meV, which is sufficiently low, so both these structures are good configurational models for Ti–12.5 at.% Mo alloys. The computational equivalent of a real binary solid solution can be considered to be different arrangements of degenerate domains of different ordered structures. However, in this study we consistently use structures closest to the convex hull, and these structures may be considered as the best energetic models to represent our binary systems.

**Table 1:** CE predictions of stable β-phase structures for Ti–W binary system at various compositions.

	β-phase	Atom	Site	x	y	z
Ti-8.33 W		Ti1	2m	1/6	0	1/12
		Ti2	2n	1/3	1/2	1/6
		Ti3	2m	1/2	0	1/4
		Ti4	2n	2/3	1/2	1/3
		Ti5	2m	5/6	0	5/12
		Ti6	1f	0	1/2	1/2
		W	1b	0	1/2	0
Ti-12.5 W		Ti1	8c	1/4	1/4	1/4
		Ti2	6b	0	1/2	1/2
		Mo	2a	0	0	0
Ti-16.6 W		Ti1	2e	5/6	1/4	19/24
		Ti2	2e	1/6	1/4	7/24
		Ti3	2e	1/2	1/4	5/8
		Ti4	2e	1/6	1/4	38/83
		Ti5	2e	1/3	1/4	23/24
		W	2e	1/2	1/4	1/8

The blue and green atoms are Ti and W respectively.

**Table 2:** CE predictions of stable β-phase structures for the Ti–Mo binary.

	β-phase	Atom	Site	x	y	z
Ti-8.33Mo		Ti1	8j	5/6	1/4	1/6
		Ti2	4i	1/6	0	1/3
		Ti3	4h	0	1/4	1/2
		Ti4	4i	1/2	0	2/3
		Ti5	2b	0	1/2	0
		Mo	2a	0	0	0
Ti-12.5Mo		Ti1	8c	1/4	1/4	1/4
		Ti2	6b	0	1/2	1/2
		Mo	2a	0	0	0
Ti-16.6Mo		Ti1	8f	5/6	3/8	11/12
		Ti2	8f	1/6	1/8	1/12
		Ti3	4e	0	3/8	1/4
		Mo	4e	0	7/8	1/4

The blue and green atoms are respectively Ti and Mo.

**Table 3:** CE predictions of stable β-phase structures in the Ti–V binary.

	β-phase	Atom	Site	x	y	z
Ti-12.5 V		Ti1	4i	5/6	0	1/12
		Ti2	4i	29/30	0	1/6
		Ti3	4i	1/10	0	1/2
		Ti4	4i	17/73	0	5/6
		Ti5	4i	15/41	0	1/6
		Ti6	2d	0	1/2	1/2
		Ti7	4i	42/97	0	5/6
		Ta	4i	0.3	0	1/2
Ti-25 V		Ta	4i	0.3	0	1/2
		Ti1	8c	1/4	1/4	1/4
		Ti2	4b	1/2	1/2	1/2
		V	4a	0	0	0
Ti-37.5 V		Ti1	4g	0	3/4	0
		Ti2	4h	0	3/8	1/2
		Ti3	2a	0	0	0
		V1	4h	0	7/8	1/2
		V2	2d	1/2	0	1/2

The blue and green atoms are respectively Ti and V.

**Table 4:** CE predictions of stable β-phase structures in Ti–Ta binary.

	β-phase	Atom	Site	x	y	z
Ti-12.5Ta		Ti1	4i	5/6	0	1/12
		Ti2	4i	29/30	0	1/6
		Ti3	4i	1/10	0	1/2
		Ti4	4i	17/73	0	5/6
		Ti5	4i	15/41	0	1/6
		Ti6	2d	0	1/2	1/2
		Ti7	4i	42/97	0	5/6
		Ta	4i	0.3	0	1/2
Ti-25Ta		Ti1	8c	1/4	1/4	1/4
		Ti2	4b	1/2	1/2	1/2
		Ta	4a	0	0	0
Ti-37.5Ta		Ti1	2i	7/8	0	1/8
		Ti2	2i	15/16	1/4	9/16
		Ti3	1c	0	1/2	0
		Ti4	2i	1/4	1/2	3/4
		Ti5	2i	7/16	0.25	1/16
		Ti6	1h	1/2	1/2	1/2
		Ta1	2i	3/16	1/4	5/16
		Ta2	2i	5/16	3/4	3/16
		Ta3	2i	3/8	0	5/8

The blue and green atoms are respectively Ti and Ta atoms.

**Table 5:** CE predictions of stable β-phase structures in Ti–Nb binary.

	β-phase	Atom	Site	x	y	z
Ti-12.5Nb		Ti1	8c	1/4	1/4	1/4
		Ti2	6b	0	1/2	1/2
		Nb	2a	0	0	0
Ti-25Nb		Ti1	4e	1/2	1/4	3/4
		Ti2	2a	0	0	0
		Nb	2a	0	0	1/2
Ti-37.5Nb		Ti1	4k	0	0	3/4
		Ti2	4I	0	1/2	7/8
		Ti3	2d	0	0	1/2
		Nb1	2a	0	0	0
		Nb2	4I	0	1/2	3/8

The blue and green atoms are respectively Ti and Nb.

### β → ω transformation

After identifying the various ground state structures for the binary Ti–X β alloys using CE calculations, we investigated the effect of W, Mo, V, Ta, and Nb on the β → ω transformation, which is predicted to occur via an ordered displacement wave initiated by the softening of the **q** = (2/3,2/3,2/3) wave vector. This corresponds to the displacement of a pair of (111) atomic planes of β relative to each other causing their collapse to form a (0002) plane of ω but leaving the next plane unchanged. This process continues by collapsing the next pairs consecutively. There are four independent <111> directions within the bcc lattice triggering this transformation. However, our calculations chose a single <111> variant to simulate the transformation due to the difficulty in creating a supercell possessing the orientation and periodicity of all the four variants. This should, however, not affect the overall energetics of β → ω transformation.

**Figure 2:**
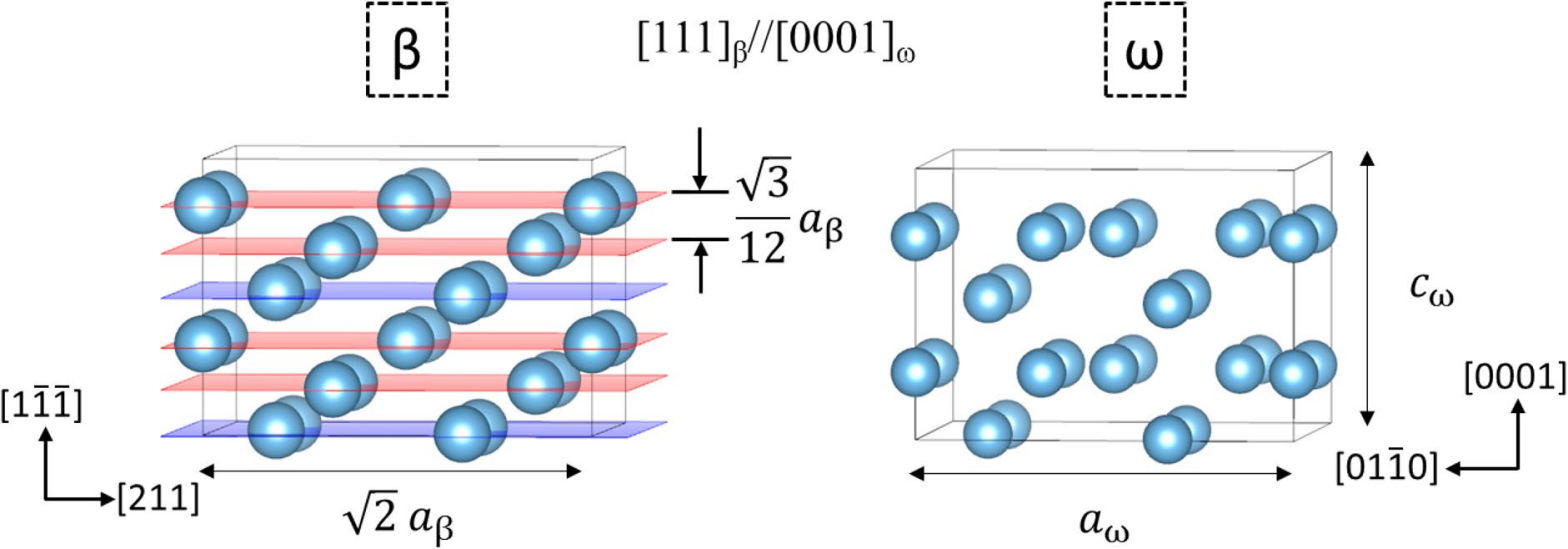
Shows the {111}_β_ plane collapse and the orientation relationship between β and ω.

The β and ω phases share a common trigonal symmetry respectively along their [111] and [0001] directions. This allows the creation of a common bcc supercell oriented along [111] direction to facilitate the manual displacement of (111) planes for the transformation simulation. The relaxed supercell model with this orientation for both β and ω phases are illustrated in Fig. 2, which also shows the collapse of {111}_β_ planes during the β → ω transformation. The $\overrightarrow{a}$, $\overrightarrow{b}$, and $\overrightarrow{c}$ lattice vectors of β-phase supercell are oriented along [011], [211] and [0 $\overline{1 }\overline{1 }$] respectively. The lattice constants for the relaxed β-phase with this orientation are 4.59, 7.95, and 2.82 Å for a, b, and c respectively. The (111) plane collapses along the $\overrightarrow{c}$ lattice vector during transformation, and the magnitude of the complete collapse is ±${a}_{\beta }\sqrt{3}$/12 that is half the spacing between two consecutive (111) planes. The atomic positions are (0, 0, 0), (2/3,1/3,1/2 + ȥ), and (2/3, 1/3, 1/2 – ȥ), where ȥ for bcc (β) and hexagonal (ω) are respectively 1/6 and 0. The next step ensures that each ground state structure obtained from CE is oriented as in Fig. 2. Lastly, the ground state structures were increased to 48 atom supercells and minimized by varying cell volume and ionic positions.

**Figure 3:**
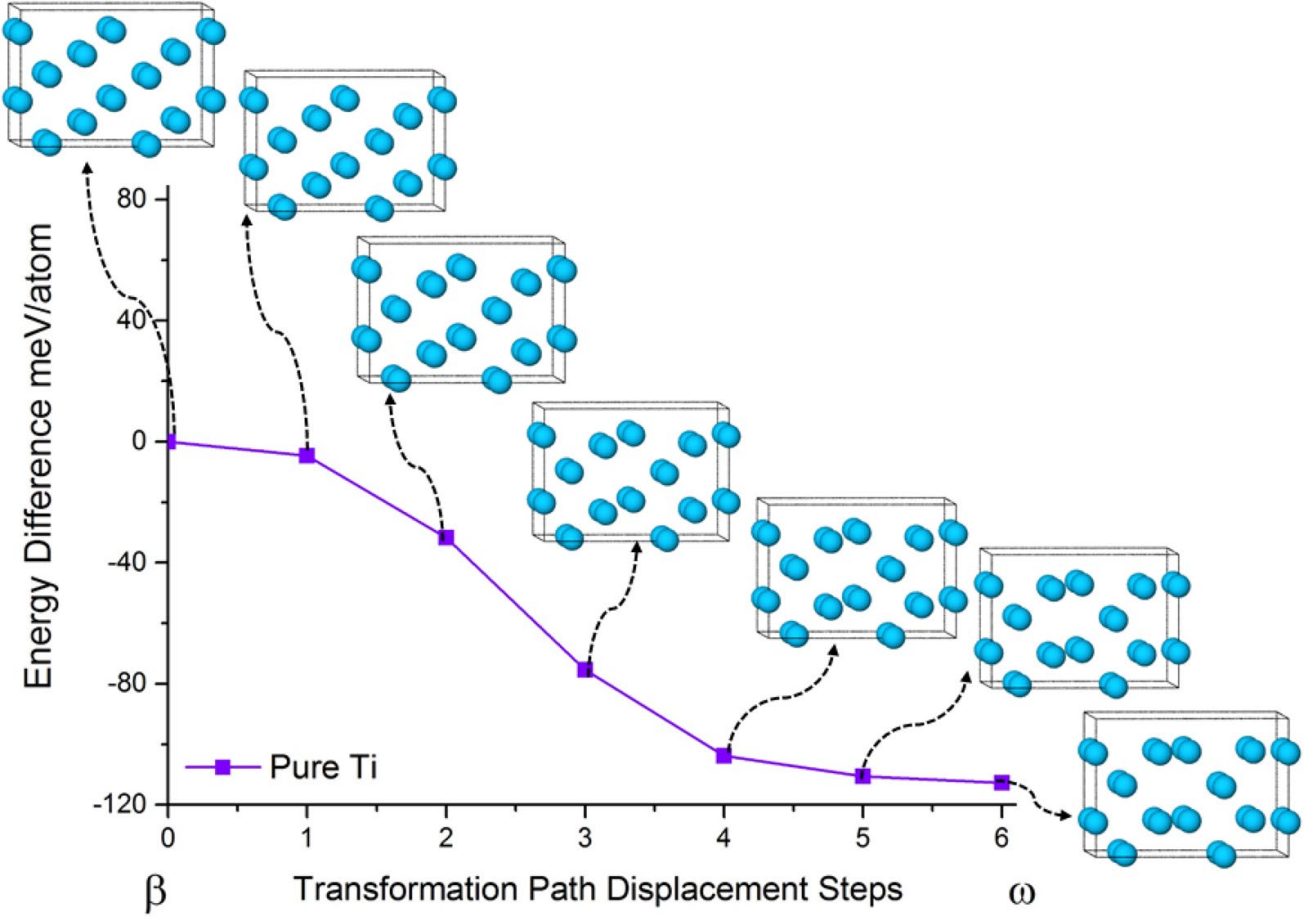
The intermediate images along the β → ω MEP in pure Ti and their energies are shown.

Figure 3 shows the images along the MEP pathway β → ω transformation for pure Ti. There is no energy barrier for this transformation, which is in agreement with prior theoretical and experimental results predicting the higher stability of ω-phase for pure Ti at T = 0 K^22,37,38^. It should be noted that the absence of an energy barrier here does not mean that this transformation should occur because its driving force is high. The absence of vibrational entropy at T = 0 K may stabilize the β-phase. In addition, other martensitic phases such as αʹ and α″ may coexist with ω-phase which impose elastic constraints on β → ω transformation and raise the energy barrier. However, the MEPs in this study help us understand the β → ω transformation mechanism.

The dynamic instability of β-phase for group IV transition metals such as Ti, Zr, and Hf at T = 0 is attributed to the presence of imaginary frequencies in their phonon spectra corresponding to the lattice instability against specific atomic movement that causes phase transformation to more stable phases^39^. The transition is also related to an elastic instability that is confirmed by negative values of the tetragonal shear modulus Cʹ = (C_11_-C_12_)^8,40^. However, the stability of bcc β-Ti increases through transfer from the s and p electrons, and this charge transfer increases the number of d-electrons by applying pressure^41^, or by alloying with transition metals such as W, Mo, V, Ta, and Nb^8,42^. A valence electrons per atom ratio (e/a) of 4.24 was also proposed as a critical number to stabilize the β-phase^8^.

**Figure 4:**
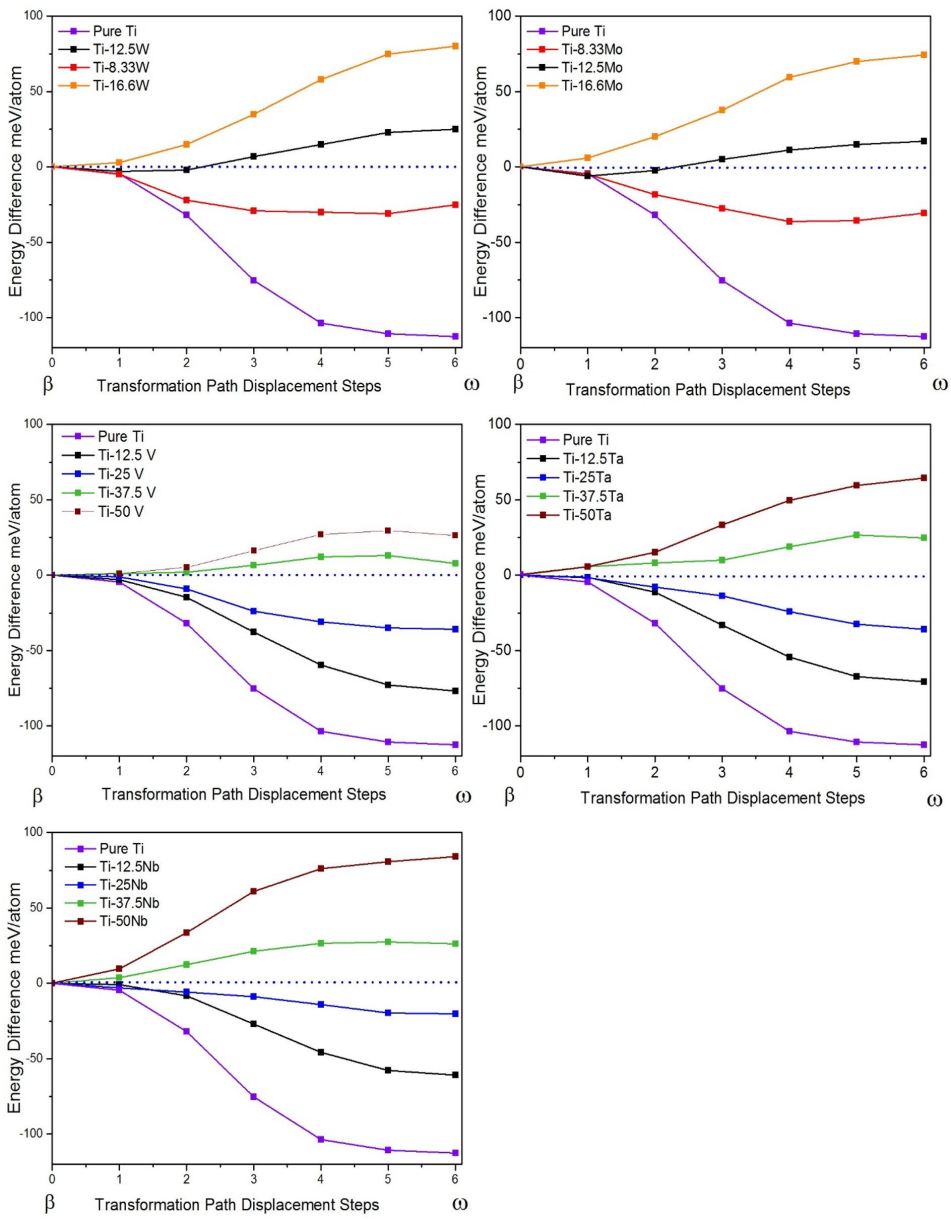
MEPs of β → ω transformation for Ti-X (X = W, Mo, V, Ta, and Nb) systems.

Figure 4 shows the MEP for the β → ω transformation in various Ti-X binary systems (X = W, Mo, V, Ta, Nb) and are compared with pure Ti. For all systems it can be seen that the stability of β-phase increases with increasing the solute content, which means that the omega transformation is progressively suppressed, and is in agreement with other theoretical and experimental results on the β stabilization by W, Mo, V, Ta, and Nb^17,42^. However, the stabilization rate of β-phase with increasing solute content is not the same for these elements. The stabilization rate of W and Mo is higher than V, Ta, and Nb. For example, 12.5 at.% Mo is required to suppress ω-phase formation in Ti-Mo system, while a concentration of more than 25 at.% Nb is required to suppress the transformation in Ti-Nb system. The higher stabilization rate of β-phase for W and Mo can be attributed to the high stability of their bcc structures in the pure form as suggested in a previous study^17^. Also, W and Mo belong to group VIa family and have more valence electrons, which increase the e/a ratio more than group Va elements V, Ta, and Nb. Clearly, the electronic structure effects, especially bonding, between Ti and β-stabilizing elements have implications for the phase stability in Ti alloys as will be shown later in this work.

### Calculations of ω-phase elastic constants

Table 6 shows the calculated elastic constants of ω-phase for pure Ti and Ti–X binary alloys (X = W, Mo, V, Ta, and Nb). These single crystal elastic constants for pure Ti agrees well with previous calculations^43,44^ and finite temperature experiments^21^. Also, the calculated polycrystalline bulk modulus *B*, shear modulus *G*, and Young’s modulus *E* of pure Ti ω-phase agree well with literature values^21,43,44^. Unlike the β-phase in pure Ti whose *c*_*11*_ - *c*_*12*_ < *0* indicating instability^45^, the ω-phase in pure Ti satisfies the mechanical stability criteria: *c*_*11*_ >|*c*_*12*_|, *c*_*33*_*(c*_*11*+_ *c*_*12*_*)* > *2* ${c}_{13}^{2}$, *c*_*11*_ > ${c}_{13}^{2}$, and *c*_*44*_ > *0*. This confirms the possibility of retaining metastable ω-phase at low temperatures.

**Table 6:** The elastic constants of ω-phase in Ti–X binary systems (X = W, Mo, V, Ta, and Nb) at 0 K obtained using first-principles calculated.

Alloys	*C*_*11*_	*C*_*12*_	*C*_*13*_	*C*_*33*_	*C*_*44*_	*C*_*66*_	*B*	*G*	*E*	Reference
Ti	193.7	77	51	248	54	58	113	62	159	
Ti	192.3	77	54.6	248	54.3	57	115	61	155	^44^ ab initio
Ti	194	81	54	245	54	56	118	63	158	^43^ ab initio
Ti	179	90	60	228	70	44	111	60	152	^21^ Expt
Ti–8.33 W	202	58	100	212	42	41	119.2	52	138	
Ti–12.5 W	204	101	69.5	235.1	37.2	52	125	50	132	
Ti–16.6 W	209	118	65	245	36	45	129	148	128	
Ti–25 W	211	127	74	240	25	41	135	40	110	
Ti–8.33 Mo	294	133	186	305	46	45	206	61	161	
Ti–12.5 Mo	301	187	138	357	38.8	57	209	55	152	
Ti–16.6 Mo	312	211	146	338	20	50	218	43	116	
Ti–25 Mo	318	157	157	317	− 34	6	225	− 6	− 21	
Ti–8.33 V	235	53	57	196	55	50	111	58	149	
Ti–12.5 V	198	90	62	230	47	54	117	56	145	
Ti–16.6 V	196	115	61	251	46	40	124	51	135	
Ti–25 V	192.2	68.2	130	188	39	40	129	42	115	
Ti–37.5 V	186.9	101	98	191	50.2	37	130	44	113	
Ti–50 V	186	122	96	193	− 16	43	132	10	21	
Ti–8.33 Ta	196	99	57	248	50	48	118	56	145	
Ti–12.5 Ta	192	105	58	247	48	43	119	53	139	
Ti–16.6 Ta	196	115	61	251	46	39	124	51	135	
Ti–25 Ta	192	68	130	188	39	40	129	42	115	
Ti–37.5 Ta	264	77	74	202	26	26	140	35	98	
Ti–50 Ta	267	83	76	213	27	18	146	31	89	
Ti–8.33 Nb	196.5	89.86	58.3	233.6	47.2	53	115	56.6	148.3	
Ti–12.5 Nb	188	71	89	180	48	45	117	53	138	
Ti–16.6 Nb	147	131	74	222	39	49	119	45	105	
Ti–25 Nb	199	71	106	192	30	29	123	43	114	
Ti–25 Nb	226	116	80	276	54	38	140	32	86	^4^ ab initio
Ti–25 Nb	162	124	84	234	22	18	127	27	75	^22^ ab initio
Ti–37.5 Nb	169	106	99	177	31	42	125	35	97	
Ti–50 Nb	192	147	79	237	2.64	17	137	16	46	

The single crystal elastic constants c_ij_, bulk modulus *B*, shear modulus *G*, and Young’s modulus *E* are all in GPa.

The elastic constants (c_11,_ c_12_, c_13_, and c_33_) of binary Ti–*X* alloys show no clear trend with increasing β-stabilizer content. However, both *c*_*44*_ and *c*_*66*_ decreases with increasing β-stabilizer content. It should be noted that in addition to calculating the elastic constants of the concentrations described in β → ω transformation section, we included the 25 at.% concentration for W and Mo, and 8.33 at.%, 16.6 at.% for V, Ta, and Nb using the same approach of getting the ground state structures obtained from the CE results. The study of these additional concentrations is for consistency and the completeness of the current work.

**Figure 5:**
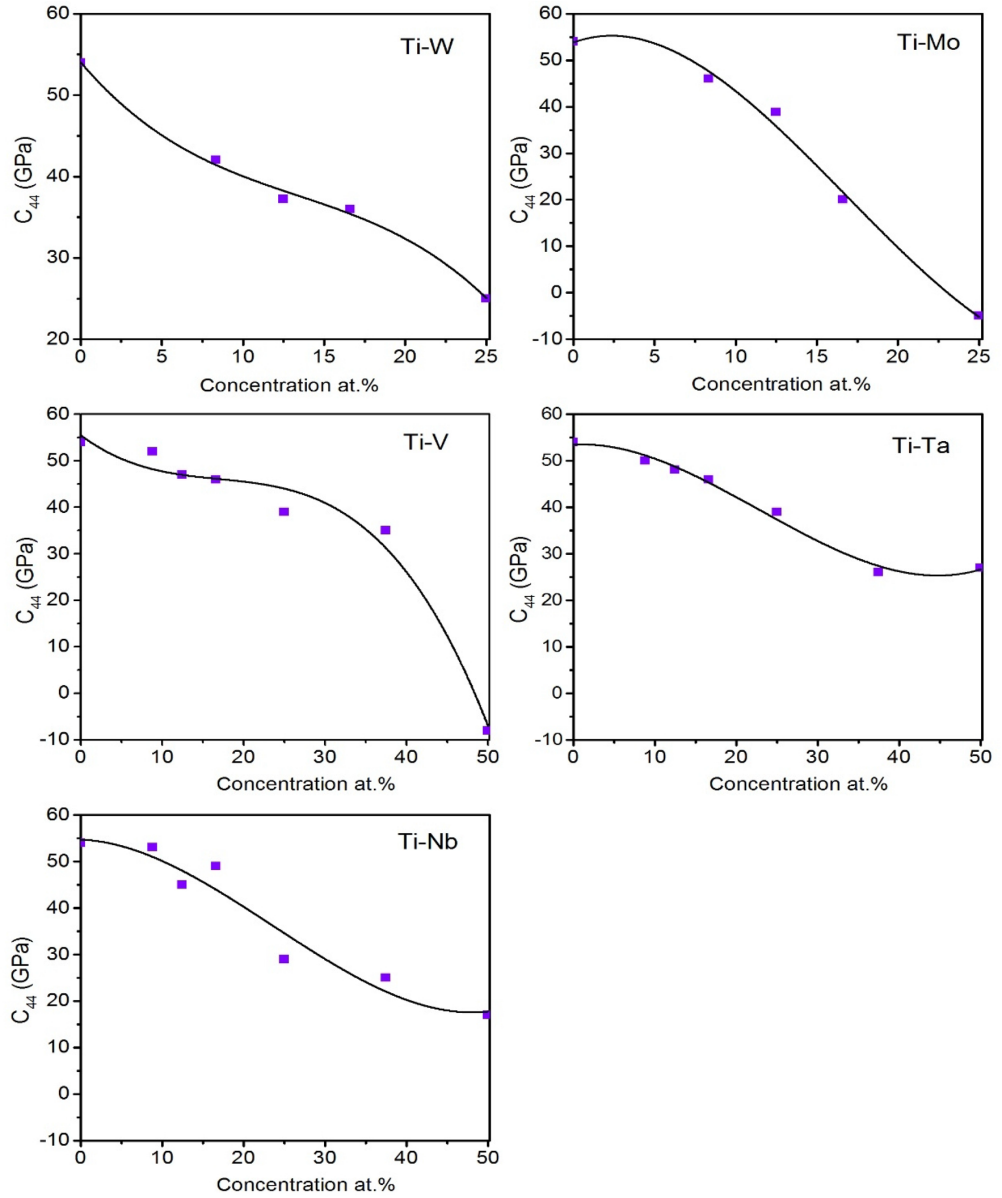
Calculated *C*_*44*_ elastic constants of the ω-phase Ti-X binary systems (X = W, Mo, V, Ta, and Nb).

The *c*_*11*_ and *c*_*12*_ elastic constants for Ti–W and Ti–Mo systems increase while *c*_*13*_ decreases with increasing concentration. However, this trend could not be observed for Ti–V, Ti–Ta, and Ti–Nb systems. Interestingly, for all systems studied here, the *c*_*44*_ elastic constant decreases with increasing concentration indicating the mechanical stability of ω-phase decreases with increasing alloying content, which agrees with our predicted MEPs in Fig. 4. Also, *c*_*66*_ first decreases then increases with concentration for Ti–W, Ti–Mo, and Ti–V systems, while it monotonically decreases with increasing concentration for Ti–Ta, and Ti–Nb systems. Figure 5 shows *c*_*44*_ elastic constants of binary Ti–X alloy studied here. The sharp decrease in *c*_*44*_ values for Ti–Mo, Ti–W, and Ti–V with increasing alloy concentration to some specific point is related to the instability of ω-phase at that concentration. We can relate this response to MEPs in Fig. 4 where the energy of ω-phase is higher than β-phase energy. The same is true for Ti–Ta and Ti–Nb systems. Also, this elastic instability sometimes manifests itself with negative values of elastic constants such as in the *c*_*44*_ of Ti–Mo and Ti–V systems. Therefore, realistic elastic constants values should be considered within the concentration range of ω-phase stability, i.e. less than 12.5 at.% for (Mo, W), and 25 at% or less for (V, Ta, and Nb). This was confirmed experimentally before^38,46^.

To the best of our knowledge the only available calculated elastic constants for ω-phase, to compare our results with, are for Ti–25 at.% Nb reported by Lazer et al.^4^ and Sun et al.^22^, and these values are listed in Table 6. Both studies used different structural models and some differences between calculated results for these two studies are expected. Our structural model for ω-phase with 25 at.%Nb is based on the cluster expansion, which is different from the previously used structural models, so some difference between our results and previously reported data is also expected.

**Figure 6:**
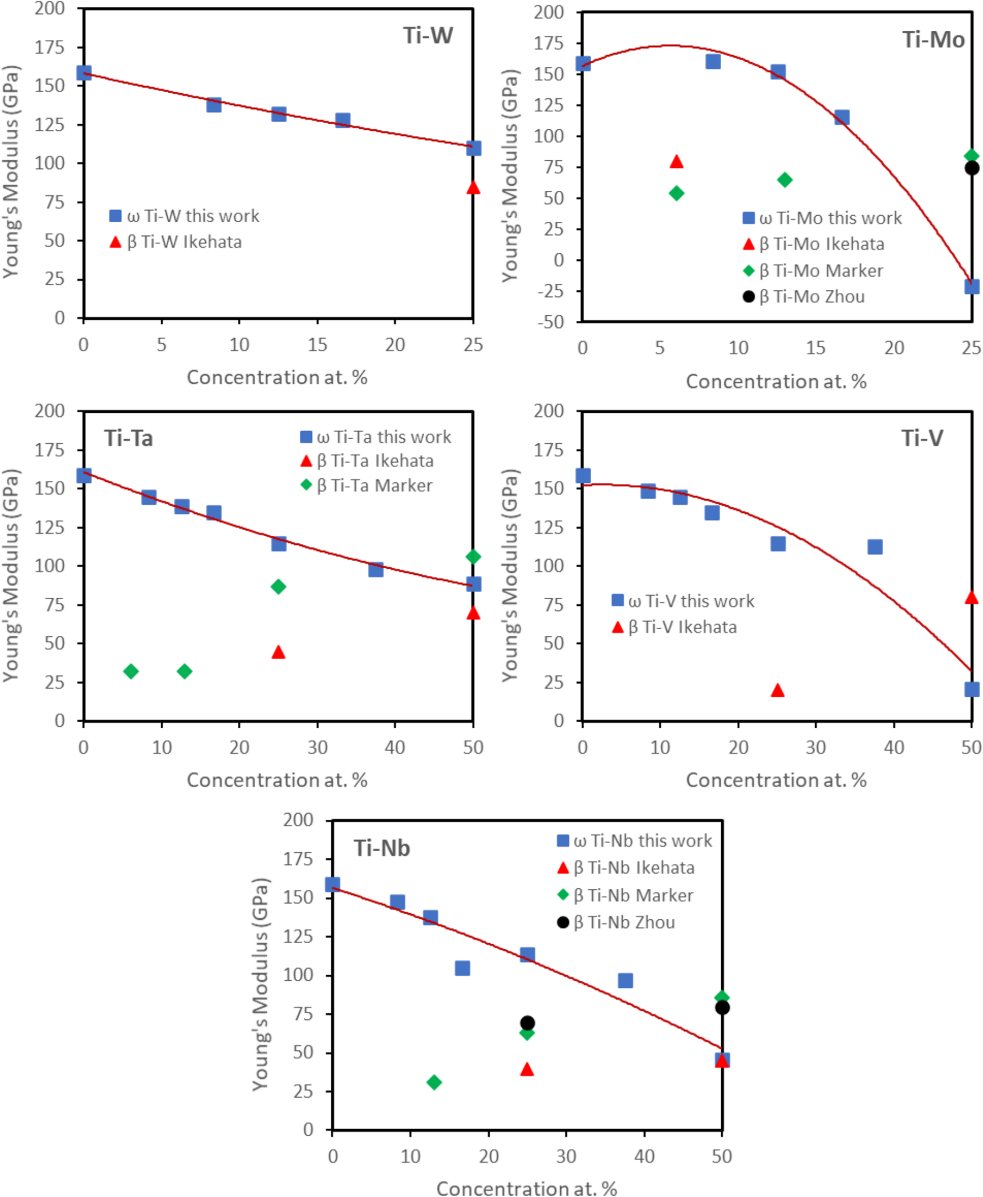
Calculated Young’s moduli of the ω-phase Ti-X binary systems (X = W, Mo, V, Ta, and Nb). Previous available calculated Young’s moduli values for β-phase are also included for comparison^8,18,19^.

Figure 6 summarizes polycrystalline Young’s moduli we calculated for Ti–X alloys. All β-stabilizers explored here decrease the Young’s moduli of ω-phase with increasing concentration. However, if we consider the stable concentration range for ω-phase, which is below 12.5 at.% for W and Mo and 25 at.% for V, Ta, and Nb, we note that at 12.5 at.% concentration of W and Mo, the Young’s modulus decreases by 17% and 5% respectively. However, a decrease of ~ 28% can be observed for V, Ta, and Nb with 25 at.% concentrations. Also, the shear modulus *G* decreases by ~ 15% for W and Mo, while a significant 32% decrease is seen for V, Ta, and Mo. The present calculations confirm that ω-phase has higher elastic moduli compared to metastable β and α″ phases^8,47^.

The composition of as-quenched (athermal) ω-phase is different from that of isothermal ω. The isothermal ω is obtained after aging below β-phase solvus and results from elemental partitioning of β-stabilizers during aging heat treatment^15,48^. Although it has not been shown before that changing the chemical composition of ω-phase directly affects its mechanical properties, the compositional partitioning between β and ω phases is known to affect the deformation mode in Ti-Mo alloys^13^. Also, another study found that the high difference between the shear modulus of β and ω phases can suppress the TRIP and TWIP effects^12^. In fact, ω-phase with its large shear modulus obstructs the {1 $\overline{1 }$ 0}_β_ < 110 > _β_ shuffle movement required to facilitate the β → α̋ martensitic transformation, which limits the TRIP effect. Our calculations suggest that the as quenched (athermal) ω-phase with high concentration of β-stabilizers can better enhance the elastic properties of Ti alloys than the solute-depleted ω-phase whose shear modulus is higher. Therefore, we argue that a thermomechanical treatment procedure that limits partitioning of β-stabilizers within the ω-phase should be considered when designing a high strength Ti alloys with good ductility. Such an insight has not been envisaged before because the ω-phase was considered to be intrinsically brittle with no recourse.

**Figure 7:**
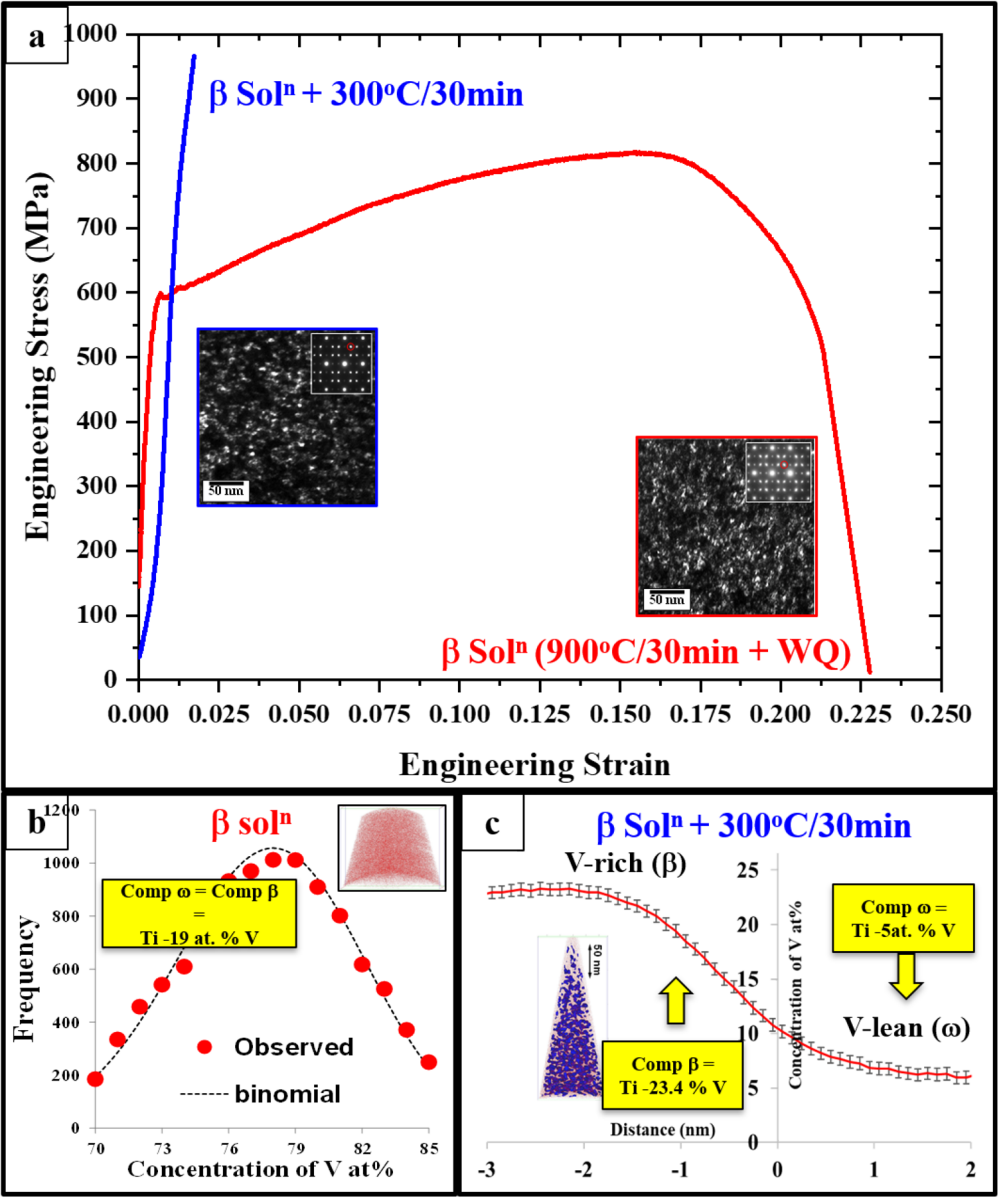
(**a**) Tensile test curves and Dark-field TEM (DFTEM) images of the β-solutionized condition (red curve) and the β-solutionized and annealed condition (blue curve). Insets show the [011]_β_ electron diffraction patterns used for DFTEMs recording, (**b**) atom probe tomography (APT) results showing the binomial distribution comparison of V concentrations in β-solutionized specimen, (**c**) shows the 1D composition profile plot for V partitioning across ω/β interface (the inset shows the isoconcentration surfaces).

**Figure 8:**
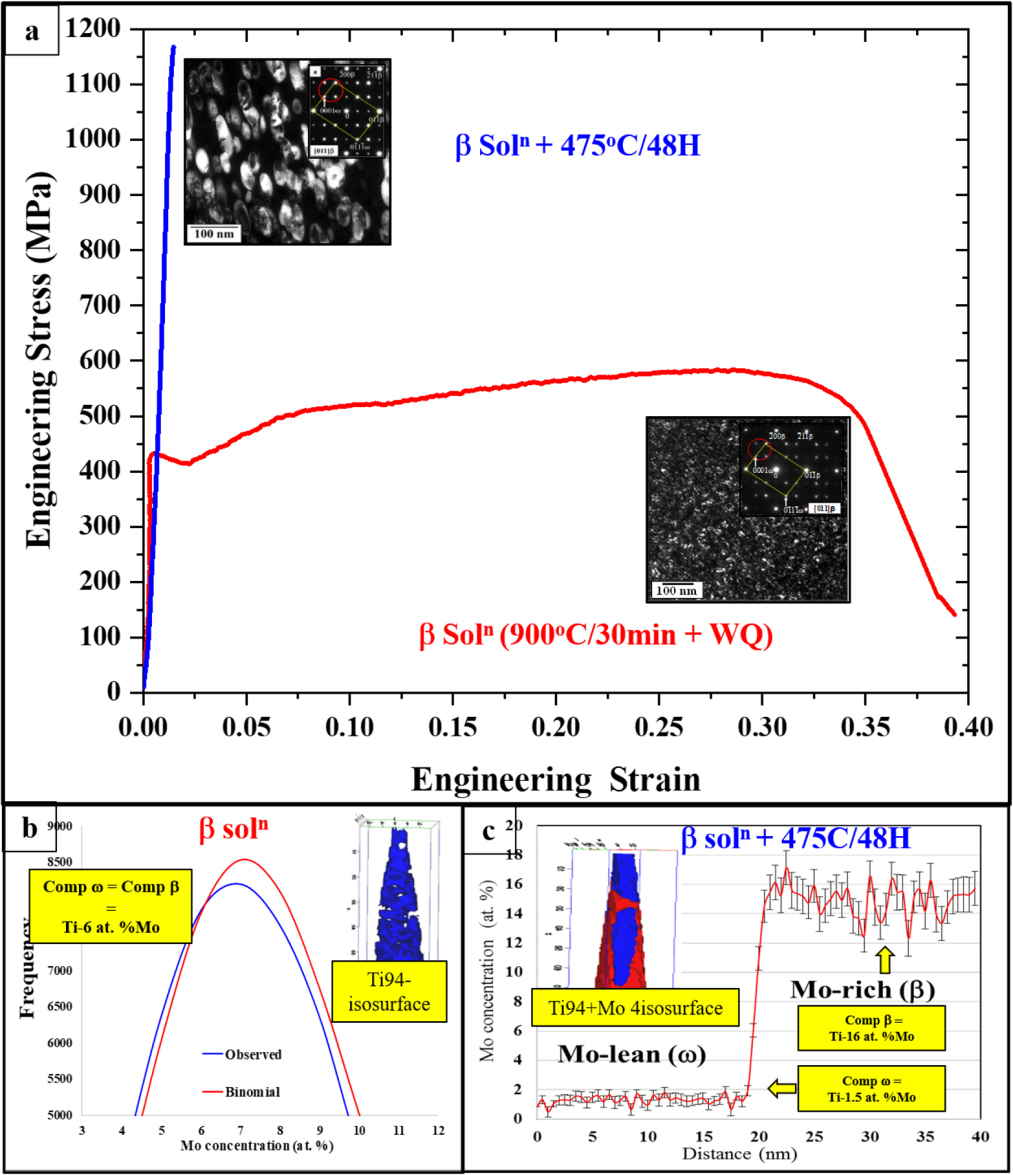
(**a**) Tensile test curves and Dark-field TEM (DFTEM) images of the β-solutionized condition (red curve) and the β-solutionized and annealed condition (blue curve). Insets show the [011]_β_ electron diffraction patterns used for DFTEMs recording, (**b**) atom probe tomography (APT) results showing the binomial distribution comparison of Mo concentrations in β-solutionized specimen, (**c**) shows the 1D composition profile plot for Mo partitioning across ω/β interface (the inset shows the isoconcentration surfaces).

Two prototype/model alloys were selected for complementary experimental investigations. These two are simple binary alloys involving two of the most commonly used β stabilizers in Ti alloys, V and Mo. The results are summarized in Figs. 7 and 8. While Fig. 7 shows the experimental results from the Ti–20 wt.% V alloy, Fig. 8 focuses on Ti–12 wt.% Mo alloy. Both these alloys form quenched-in athermal ω precipitates as well as isothermal ω precipitates.

The binary model alloy, Ti–19 at.%V (Ti–20 wt.%) was subjected to a β-solutionizing treatment, followed by water-quenching, and subsequent aging at 300 °C/30 min, below the ω-solvus temperature. The dark-field TEM (DFTEM) images of ω precipitates and tensile test results for the β-solutionized and annealed conditions are shown in Fig. 7a. The dark-field images for both conditions were recorded along the [110]_β_ zone axis, and the corresponding selected-area electron diffraction (SAED) patterns are shown as insets. Highly refined scale ω precipitates (~ 5–10 nm) were observed in the β-solutionized microstructure, and the size scale of these ω precipitates remained nearly the same (~ 5–10 nm) after annealing at 300 °C/30 min. The presence of intensity maxima at 1/3 and 2/3 $\stackrel{-}{[2}1\stackrel{-}{1]}$_β_ locations in the [110]_β_ SAED further confirms the presence of ω-phase in both conditions.

Figure 7b shows the atom probe tomography (APT) results from the β-solutionized condition. The binomial distribution plots were obtained by measuring every 100 atom block within the 3D atom probe reconstruction^49^. The V ion distribution plot (red dotted line) clearly follows the expected distribution from a random solid solution (black dotted line). This means that the V partitioning between β and ω phases was not perceptible within the limitations of this analysis technique using binomial distribution. This indicates the formation of athermal congruent ω precipitates within the β matrix. Therefore, both phases have the same composition in the solutionized condition, i.e. ω phase has a composition of Ti–19 at.% V. On the other hand, a significant partitioning of V between ω and β phases could be observed in the 300 °C/30 min annealed specimen as shown in Fig. 7c. A depletion of V is observed for the isothermal ω precipitates (~ 5 at.% V), while the surrounding β matrix contains ~ 24 at.% V. These compositions were determined from APT reconstructions, based on V profiles calculated across isoconcentration surfaces (or isosurfaces) used for delineating the ω/β interfaces.

The engineering tensile stress vs engineering strain curves for both β-solutionized, and β-solutionized and 300 °C/30 min annealed conditions are shown in Fig. 7a. A significant strain hardening with an ultimate tensile strength (UTS) of ~ 800 MPa, and a ductility of 20% could be observed for the β-solutionized condition (red curve), while the β-solutionized and annealed specimen showed much higher UTS of ~ 1000 MPa with a limited ductility of < 2% (blue curve). This substantial change in the mechanical properties can be attributed to the dependence of the ω-phase mechanical properties on its β-stabilizer content. The trends in the variation of DFT-computed Young’s modulus as a function of V content, can be inferred from Fig. 6. Based on these trends, the computed Young’s modulus for the Ti–19 at.% V and Ti–5 at.% V can be approximated to be 125 GPa and 150 GPa respectively. This large increase in the modulus of the isothermal ω precipitates (due to the rejection of V) as compared to the quenched-in athermal congruent ω precipitates, leads to the higher yield strength in case of isothermally annealed condition. This change is also accompanied by the loss of deformability of the isothermal ω precipitates, leading to loss of ductility, as experimentally observed (Fig. 7a).

Similar experimental results from the Ti–12 wt% Mo alloy, equivalent to approximately Ti–6.5 at.% Mo, have been summarized in Fig. 8. The dark-field TEM (DFTEM) images of ω precipitates and tensile test results for the β-solutionized and annealed conditions are shown in Fig. 8a. The dark-field images for both conditions were recorded along the [110]_β_ zone axis, and the corresponding selected-area electron diffraction (SAED) patterns are shown as insets. Very fine scale ω precipitates (~ 5–10 nm) were observed in the β-solutionized microstructure, while coarser ω precipitates (~ 70–90 nm), ellipsoidal in shape, could be observed after annealing at 475 °C/48H. The presence of intensity maxima at 1/3 and 2/3 $\stackrel{-}{[2}1\stackrel{-}{1]}$_β_ locations in the [110]_β_ SAED further confirms the presence of ω-phase in both conditions.

Figure 8b shows the atom probe tomography (APT) results of the β-solutionized alloy. The binomial distribution plots were obtained by measuring every 100 atom blocks within the 3D atom probe construction^49^. Mo ion distribution plot (blue line) clearly follows the expected distribution from a random solid solution (red line). This means that the Mo partitioning between β and ω phases was not perceptible within the limitations of this analysis technique using binomial distribution. This indicates the congruent formation of ω precipitates within the β matrix. In other words, both phases have the same composition in the solutionized condition. On the other hand, a significant partitioning of Mo between ω/β could be observed in the annealed specimen as shown in Fig. 8c. A depletion of Mo is observed for the coarse ω precipitates (~ 1.5 at.% Mo), while a rich Mo content of ~ 16 at.% can be observed for the surrounding β matrix. These compositions were obtained by measuring the 1D composition profile across the ω/β interface.

The engineering tensile stress vs engineering strain curves for both β-solutionized, and β-solutionized and annealed conditions are shown in Fig. 8a. A significant strain hardening with an ultimate tensile strength (UTS) of ~ 559 MPa, and a ductility of 35% could be observed for the β-solutionized condition (red curve), while the β-solutionized and annealed specimen showed much higher UTS of ~ 1070 MPa with a limited ductility of < 5% (blue curve). Similar to the previously discussed case of the Ti–19 at.% V alloy, in the case of the Ti–6 at.%Mo alloy, this significant change in mechanical properties can be attributed to changes in the Young’s modulus of the ω-phase as a function of its β-stabilizer content. Figure 6 shows a that with increasing Mo content the Young’s modulus of ω decreases. While the experimental values of compositions for the athermal congruent ω (8.33 at.% Mo) and the annealed isothermal ω (1.5 at.% Mo) may not exactly correspond to the discrete ω compositions shown in Fig. 6, it is important to note that the range of Mo compositions evaluated by the DFT computations, covers the entire experimental range of compositions. Furthermore, other additional factors such as the change in the deformation mode within the β matrix, from twinning to slip, and the size of ω-phase precipitates can also influence the mechanical response of the condition containing the isothermal ω precipitates^13^. A recent study by Lai et al.^16^ revealed severe loss of ductility in the Ti–12 wt.% Mo alloy upon short term annealing for 10 min. at 400 °C. While this short aging treatment did not result in any discernable change in the volume fraction, size, and inter-particle spacing of ω precipitates, a significant Mo rejection from the isothermal ω precipitates was reported based on APT results^21^. The study attributed this loss of ductility to a large increase in the ω shear modulus. These previously reported results further validate the DFT-computed composition-dependent elastic constants and Young’s modulus values reported in the present paper.

### Phase stability dependence on electronic structure

The basic features of the chemical bonding that stabilizes β-phase upon alloying in Ti alloys is still largely unknown. The electronic density of states (EDOS), which reveals the occupation of various energy levels, (i.e. the number of electronic states per unit energy) can shed light on this. A description of a chemical bond types as metallic or covalent can be predicted from the density of states (DOS) occupation and the pseudogap position with respect to the Fermi level *E*_*F*_. A high occupation with a pseudogap away from the *E*_*F*_ indicates metallic bonding and a lower crystal stability. A lower occupation state at the fermi level coupled with the opening of a pseudogap near *E*_*F*_ indicates a strong covalent bonding between atoms and implies a higher phase stability in metallic system^50^.

**Figure 9:**
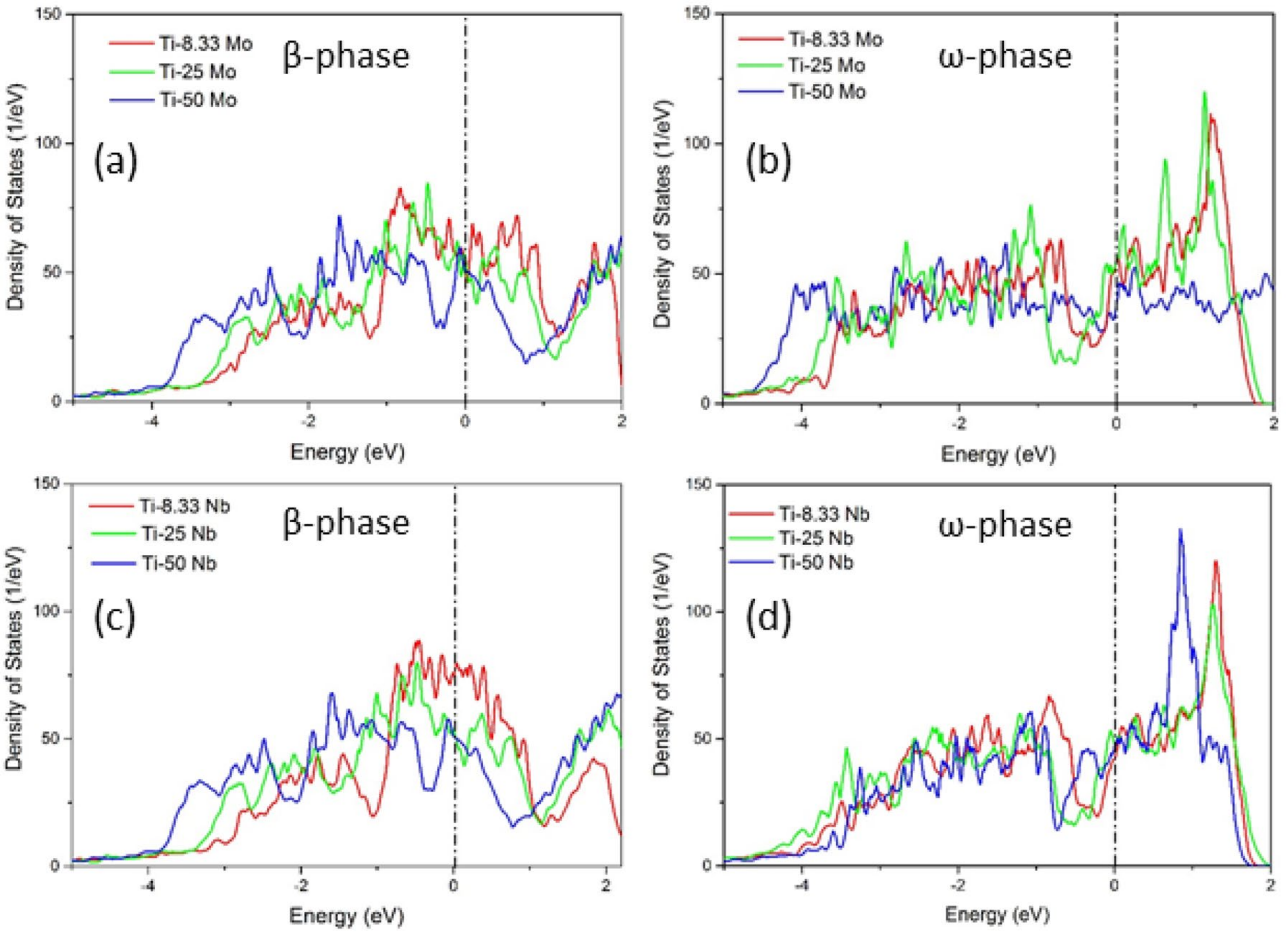
The calculated total density of states (DOS) in Ti-Mo and Ti-Nb binary alloys. (**a**, **b**) are respectively DOS of β and ω-phases in Ti-Mo. (**c**, **d**) are respectively DOS of β and ω-phase in Ti-Nb.

The β-stabilizers elements considered in this study can be divided into two groups. The VIa group elements W and Mo show a high stabilization rate at increasing concentrations. On the contrary, Va family group elements V, Ta, and Nb has a lower stabilization rate at increasing composition. Therefore, for DOS calculations, we chose Mo, and Nb as representatives of these two groups. Thus, the DOS calculations were performed for the β and ω phases of Ti–Nb and Ti–Mo systems at their equilibrium geometries. The DOS calculation results are shown in Fig. 9.

The energy levels in Fig. 9 range from − 5 to 2 eV, and the zero-energy denoted by the vertical dashed line is the Fermi level. The DOS shapes for β and ω phases in Ti–Mo and Ti–Nb are similar. This confirms the previous finding that DOS spectrum shape depends on the crystal structure rather than the elements type^8,51^. A clear pseudogap can be seen between the low-energy bonding and high-energy anti-bonding regions for β-phase of Ti–Mo and Ti–Nb binary systems as shown in Fig. 9a, c respectively. The high value of the DOS at the Fermi level, which is located in the bonding region, especially in case of lower concentrations of Mo and Nb, indicates a metallic bonding between atoms and a reduction in β-phase stability. Considering the ω-phase, the Fermi level is located very close to the pseudogap, for lower alloying concentrations in both Ti–Mo and Ti–Nb systems as shown in Fig. 9b, d, which indicates a covalent bonding character and higher stability of ω-phase with respect to β-phase. However, with increasing Mo and Nb concentration, the valence d-electrons increase and the Fermi level shifts towards the pseudogap in β-phase, which creates a stronger metallic bonding and enhances stability. Furthermore, higher solute concentration shifts the Fermi level towards the antibonding region with higher values of DOS and destabilizes the ω-phase. These DOS observations of destabilization of ω-phase with increasing concentration is consistent with our predicted MEPs and elastic constants calculations.

## Conclusion

The effect of five β-stabilizing elements W, Mo, V, Ta, and Nb on the phase stability and the elastic properties of ω-phase in binary Ti alloys were systematically studied using first-principles calculations. The CE method was utilized the find the stable atomic configurations for Ti–X alloys at various compositions. These atomic structures were later utilized to find the MEPs for β → ω martensitic transformation using the solid state nudged elastic band (SSNEB) method. A higher stabilization rate of β-phase with increasing composition was observed for Ti–W and Ti–Mo due to their higher number of valence electrons, while a lower stabilization rate was observed for Ti–V, Ti–Ta, and Ti–V systems. The compositions causing full stabilization of β-phase agree well with experimental studies. The main outcomes of the present study can be listed as follows:

- The single crystal and the polycrystalline elastic properties of the ω-phase were predicted as a function of β-stabilizers’ concentrations. In general, all β-stabilizing elements considered in this study decreased the elastic moduli of ω-phase with increasing concentration. A decrease of *E* by 17% and 5% could be observed for that Ti–W and Ti–Mo, respectively at 12.5 at.% concentration, while a decrease of ~ 28% could be observed for Ti–V, Ti–Ta, and Ti–Nb systems with 25 at.% concentration. Also, *G* decreases by ~ 15% for W and Mo and 32% for V, Ta, and Mo. An experimental Ti–6.5 at.% Mo alloy was prepared to study the mechanical properties with respect to Mo partitioning between the β and ω phases. A significant increase in tensile strength and a decrease in ductility was observed in the annealed condition due to the low content of Mo in the ω phase, while lower strength and high ductility was observed in the solutionized and quenched condition where Mo content in ω-phase was high and nearly equal to that of the β-phase.
- The present study conclusively establishes that the elastic constants and shear modulus of quenched athermal ω significantly differ from those of aged isothermal ω, and this correlates well with the observed variations in the mechanical behavior of the Ti-alloys with β + ω microstructure. The lower shear modulus of athermal ω as compared to isothermal ω implies that the bonding is weaker in case of the former, and consequently athermal ω precipitates can be more easily sheared during plastic deformation. This can enhance deformability of the alloy, and correlates well with the higher ductility reported previously for β Ti alloys containing athermal ω precipitates. On ageing, the β stabilizing solutes are rejected by the ω precipitates leading to the formation of isothermal ω precipitates. Their significantly higher shear modulus makes isothermal ω precipitates less plastically deformable and more embrittling.
- These results suggest that the negative effect of ω-phase on ductility can be remedied by following a careful heat treatment procedure, which promotes the precipitation of ω-phase with higher concentration of β-stabilizers and decreases the elastic constant of this phase. Such heat treatments could be similar to what was reported previously^14,52^. Therefore, the ω-phase may not always be detrimental like previously believed. In fact, β-stabilizers may even enhance the ductility of Ti–X alloys via tuning their elastic constants. Such insights were not available from experiments and point out that contrary to popular belief, the presence of the ω-phase in Ti alloys does not necessarily embrittle the alloy.
- The present study revealed for the first time that the nature of bonding within the w phase changes substantially as a function of content of b stabilizing element. Thus, the DFT-based computed density of states (DOS) reveals that quenched athermal ω, with a higher b stabilizer content, exhibits more non-directional metallic type bonding. On the other hand, aged isothermal ω, with a lower b stabilizer content, exhibits more directional covalent type bonding.

## Data Availability

The data used to generate the results within the current study can be obtained from the corresponding author upon request.
